# Moisture Sorption Behaviour and Mould Ecology of Trade Garri Sold in South Eastern Nigeria

**DOI:** 10.1155/2014/218959

**Published:** 2014-11-16

**Authors:** Tochukwu Samuel, J. Obeta Ugwuanyi

**Affiliations:** Department of Microbiology, University of Nigeria, Nsukka 410001, Nigeria

## Abstract

Garri is a creamy white or yellow starchy grit produced by roasting to gelatinization and dryness of peeled, washed, mashed, and fermented dewatered cassava roots. It is the most important product of cassava in West and Central Africa. Mean moisture content of yellow and white garri was 11.11% and 10.81% within 24 hrs of sampling from the market, increasing to 17.27% and 16.14%, respectively, following 3 months of storage at room temperature. The water activity of samples varied from initial 0.587 to 0.934 following storage. Moisture sorption isotherms, determined by static gravimetric techniques at 20° and 30°C, showed temperature dependent BET Sigmoidal type II behaviour typical of carbohydrate rich foods but modulated very slightly by the content of palm oil. Equilibrium moisture content decreased with increase in temperature at constant water activity. A total of 10 fungal species belonging to the genera *Mucor*, *Penicillium*, *Cephalosporium*, *Aspergillus*, *Scopulariopsis*, *Rhizopus, and Paecilomyces* were identified, with range increasing with water activity of samples.

## 1. Introduction

Cassava (*Manihot esculenta *Crantz), an important root crop and major player in the food security of producer nations, provides energy as the staple food of over 500 million people in the tropics and subtropics. It gives the highest yield of starch per hectare of any crop. Over 160 million tons of cassava is produced globally per annum, ranking it as 4th crop in worldwide production after rice, wheat, and maize [1, 2]. However, its high content of cyanogenic glycoside (linamarin) is a factor that reduces its acceptability and value as food [3]. Cassava is used as food generally following some form of detoxification, usually by fermentation, which results in breakdown and removal of linamarin [4–8]. It is the most perishable staple with a postharvest life of usually less than three days and so it must be processed to store for any considerable period.

A major product of cassava processing is garri which is widely consumed across West and Central Africa and beyond. Garri is a creamy white or yellow (if palm oil is added during fermentation or roasting) starchy grit produced by fermentation of peeled, washed, and mashed cassava roots which is then dewatered, sieved, and roasted to gelatinization and dryness. Garri has high fibre content and contains some essential vitamins [9]. It is the most cosmopolitan and popular product of cassava and is widely accepted in both rural and urban areas because it can be stored for considerable periods up to a few months and, unlike some other cassava products, has an appealing light aroma [10]. It can be consumed dry (as a snack) or soaked in cold water with a variety of accompaniments or reconstituted with hot water to form a dough which is eaten with soup, sauce, or stew as a major meal [11]. When garri is consumed as ready-to-eat snack, as a plain dry product, or with cold water, coconut, palm kernel, peanut, beans, or other accompaniments, there is no down-line critical control point and therefore it needs to come as wholesome product.

The postfrying/processing handling of garri for trade and consumption is associated with handling with bare hands; drying on bare cemented floor, mat, or basins; display in open buckets, bowls, basins, and mats at points of sale; and haulage over very long distances in various types of usually nonmoisture proof sacks and bags. These practices may exacerbate microbial contamination [12] and risks of food-borne diseases [13]. The mycoflora of food is of practical significance to producers, processors, and consumers since fungi are modifiers of chemical composition of foods [14]. Fungal contaminants of stored products are also responsible for discolouration, loss in nutritional value, production of off-odours, and contamination with mycotoxins [15, 16].

Control of moisture content during the processing of foods is an ancient method of preservation and probably humankind's first technology for extending the stability of foods. This is achieved by either removing or binding water to make foods microbiologically and chemically stable [17, 18]. The commonest limitation on the shelf life of food is microbial growth; hence, several preservation processes are aimed at achieving microbial stability of foods [19, 20]. Water activity of a foodstuff is defined as the ratio of vapour pressure of water in the food to vapour pressure of pure water at the same temperature [17, 21]. The moisture content of most foods increases curvilinearly (frequently sigmoidal) with water activity [22]. The relationship between the total moisture content and water activity of food over a range of values, under equilibrium conditions, yields a moisture sorption isotherm which gives information on the relation between the food and water [23, 24]. Knowledge of moisture sorption isotherms of dehydrated foods is valuable in solving food processing and engineering problems such as prediction of shelf life [25–28]. This has been successfully applied in the preservation of a number of dehydrated foods [29–31]. However, to our knowledge, this has not been studied in garri and a variety of dry food staples in the tropics, being also the countries most challenged by issues of food security.

This study was undertaken to determine the water activity and moisture sorption characteristics of garri and to relate these to the mould ecology of the product with a view to be added to the body of knowledge needed to achieve enhanced shelf life of this very important security food.

## 2. Materials and Methods

### 2.1. Collection of Samples

Three samples each of freshly processed (within 48 hr) white and yellow garri were randomly collected from each of five local markets: Ikpa and Ogige main markets (Nsukka Local Council Area), Nkwo Ibagwa market (Igbo-Eze South Local Council Area), and Obollo-Afor and Obollo-Eke markets (Udenu Local Council Area), all in Enugu State (a major garri producing belt), Nigeria. Samples were collected in clean and dry laboratory quality cellophane bags and taken to the laboratory for analysis within 12 hrs.

### 2.2. Determination of the Initial Moisture Content of Garri Samples

The moisture contents (M.C.) of the samples were determined by drying a weighed sample of garri to a constant weight in a mechanically ventilated oven at 105°C for 24 hrs. The samples were dried and weighed repeatedly until constant weight was achieved [32]. The percentage moisture content was derived on a dry weight basis. An average of three replicates was determined. Samples not used immediately for analyses were stored in insect proof hessian bags at room temperature in the laboratory for up to three months and analysed again for moisture content. Relative humidity data for the periods and study area were sourced from appropriate government agencies.

### 2.3. Determination of Water Activity of Samples

Water activity of the samples was determined by methods described by Landrock and Procter [33]. Samples (in Petri dishes) were equilibrated against appropriate saturated salt solutions at 20°C and 30°C. Both temperatures were selected as guide to reflect low nighttime temperatures obtainable during cold months of November–January/July–September and mean daytime indoor temperatures, respectively, across much of Nigeria's savanna and rainforest. Routine storage temperature of the product will mostly obtain between both temperatures. The quantity of garri sample (10 g) used was small enough not to influence the saturation behaviour of the salt solution. Water activity of the samples was determined by recording data on water loss or gain per gram of sample. The amount of water gained or lost by test samples maintained at different equilibrium relative humidity levels was plotted against the water activity of salt solution. The plot which intersects with the line representing zero moisture content represents the water activity of the sample. Nine saturated salt solutions were selected as equilibrating solutions. These include CH_3_COOK, MgCl_2_, K_2_CO_3_, NaNO_3_, NaBr, NaCl, KCl, BaCl_2_, and K_2_SO_4_ providing constant relative humidity environments ranging from 20% to 97% [34]. A 200 mL volume of each individual equilibrating solution with excess salts crystals was provided at the base of desiccator in triplicates to provide the required ranges of water activity. The volume of salt solution was selected in such a way that the amount of moisture absorbed or lost by the sample did not change the state of saturation of the salt. The salts were left to equilibrate with the atmosphere of the desiccators overnight before introduction of samples.

### 2.4. Preparation of Mould Isolation/Growth Media

Potato dextrose agar (PDA) and sabouraud dextrose agar (SDA) (Oxoid) were prepared according to manufacturer's instruction. Malt extract agar (MEA) was prepared as described by David et al. [35] while modified Czapek Dox agar (CDA) containing 20–40% sucrose as modifiers of moisture content was prepared as described by Pitt and Christian [36] and Smith [37]. The water used to prepare the medium was modified to the required water activity level according to Medina and Magan [38] and Bekada et al. [39].

### 2.5. Isolation of Moulds

Ten grams of each sample (initial and following three months storage in the laboratory) was aseptically weighed into 90 mL of 0.1% (w/v) sterile peptone water in a sterile 500 mL beaker and allowed to stand for 5 minutes with occasional stirring using a magnetic stirrer. Thereafter, 10-fold serial dilutions of samples were made and 0.1 mL aliquot of each dilution was plated on PDA supplemented with 50 *μ*g/mL of chloramphenicol. Inoculated plates were incubated in transparent airtight jars containing flasks of saturated salt solution of water activity approximately corresponding to that of the media and the sample being incubated. Controlled water activity solutions were prepared according to the data and methods of Robinson and Stokes [40]. Grains of garri samples were also directly sprinkled on media and incubated alongside the dilute samples. Plates were observed daily for growth and discarded after 28 days if no growth occurred. Colonies that developed were purified by repeated pin point inoculation on similar media and then stored on CDA at 4°C in the refrigerator until identified [41].

### 2.6. Identification of Mould Isolates

Mould isolates were identified based on cultural characteristics on SDA, CDA, and MEA. Light microscopy of isolates was performed following slide culture on SDA, CDA, and MEA and samples were stained where necessary using lactophenol cotton blue. The fungal isolates were identified based on examination of the conidial heads, phialides, conidiophores, and presence of foot cells or rhizoids. Identification took account of growth rate of isolates measured as colony diameter in 3, 5, and 7 days on SDA, CDA, and MEA at appropriate temperatures [42, 43]. Identification was achieved by using the keys of Onions et al. [44]and Raper and Fennell [45].

### 2.7. Water Sorption Characteristics and Mould Isolation

The water sorption isotherms were determined gravimetrically by exposing garri samples to atmospheres of known equilibrium relative humidities [34]. The airtight glass desiccators were placed in an incubator at 20° or 30°C for 12 hrs to allow the interior to equilibrate. Two portions of 10 g quantity each of garri (predried in desiccators with silica gel for 4 days) in open 5 cm diameter petri dishes were placed on a plastic platform inside the desiccators. At 72 hr intervals, the samples were removed and weighed until the mass remained constant for three successive times. Equilibrium moisture content of the samples at the water activity point was determined by drying one portion to a constant weight in an oven at 105°C for 24 hrs. The volume of the solution used was large enough so that moisture lost or gained by the sample being conditioned did not alter the composition of the controlling solution. Following equilibrium weight, one portion of 10 g was stored airtight in screw capped sample bottles at room temperature for three months and inoculated onto appropriate plates as above for mould isolation.

## 3. Result and Discussion

The moisture content and water activities of samples taken from the major produce markets are as shown in Tables 1 and 2 for white and yellow garri, respectively. The samples were collected during the months of March-April, when the mean daytime relative humidity was approximately 85%. After 3 months of storage in hessian bags, the samples were analyzed in the middle of the rain season, during the months of July-August during which the average relative humidity had increased to daytime mean of 91%. The mean moisture content of white garri increased from the minimum of  10.00% (at time of collection) for samples taken from Ikpa market to a maximum of 18.07% (following storage) obtained in samples from Obollo-Eke. The minimum mean increase in moisture (31.29%) was obtained in samples from Obollo-Afor while the maximum (68.41%) was obtained in samples from Obollo-Eke. For yellow garri, the minimum initial moisture content (9.77%) was obtained in samples taken from Ikpa while the highest (12.13%) was recorded for samples from Obollo-Afor. Following storage for three months, moisture content increased to a maximum of 17.93% in sample taken from Obollo-Eke. However, the highest percentage increase (80.83%) occurred in samples taken from Ikpa while, like the white samples, the minimum increase was obtained in samples from Obollo-Afor (35.45%).

The minimum increase in moisture content of white garri occurred in the sample with the highest initial moisture. Also, for yellow garri, the sample with the highest initial moisture content adsorbed the least while that with the least initial moisture content adsorbed the highest. This trend relating hygroscopy of garri to initial moisture content may be of interest for shelf life of the product if it is not to be stored in moisture proof or in modified atmosphere. Processing garri to complete dryness may thus turn out to be counterproductive for long-term storage.

The result of this study shows that the moisture content of garri at point of sale in Nsukka markets falls in the tolerable level for dry trade foods [46]. Despite being higher, in some cases, than the codex specification of 12.0% moisture for garri, it is within the safe levels (12.7% white; 13.60% yellow) reported by Oyeniran [47] who also reported comparable moisture levels in garri samples taken from Ibadan Nigeria. There were no significant differences (*P* > 0.05) in the initial moisture contents of all the garri samples obtained from 5 markets in Nsukka zone, a major garri producing belt of Nigeria. It is also in the range of moisture content recommended for garri produced by local farmers and which are not packed in any specialized containers or atmosphere but traded in nonmoisture proof bags and basins. Halliday et al. [48] and Opadokun [49] reported lower moisture contents in Kano, Northern Nigeria. This is understandable given the low atmospheric relative humidity in Kano compared to rain forest towns of Nsukka or Ibadan. The moisture content of any produce will depend on factors such as location, season, and the method of processing [50]. However, in spite of the low initial moisture content of garri the moisture sorption behavior as shown in this work suggests that alone the absolute moisture content of this food may not be indicative of qualification for long-term storability if the environment (atmosphere) is not to be modified.

The hessian and jute bags commonly used for storage of garri are not moisture proof or airtight and are therefore unsuitable for the long-term storage of this hygroscopic product. Garri packaged in hessian bags and stored in a humid atmosphere can absorb moisture to a level sufficient for the growth of fungi. This is supported by the high level moisture content obtained following storage of samples for 3 months in the laboratory at room temperature (Tables 1 and 2). Mean moisture contents of 16.14% and 17. 27% for white and yellow garri, respectively, were obtained after 3 months of storage. This was much higher than the “safe” level of 12.7–13.6% proposed by Halliday et al. [48]. Ogugbue and Obi [51] reported similar moisture content for samples collected at Port Harcourt, a humid part of Niger Delta in Nigeria. Although differences in the moisture content between white and yellow garri were not significant, the observed difference may be attributed to the modulatory effect of lipid (palm oil additive) on the moisture sorption behavior of starchy food. Moisture sorption behaviour of lipid rich foods differ from that of carbohydrate or starch rich ones [26, 28, 29, 52, 53].

**Figure 1:**
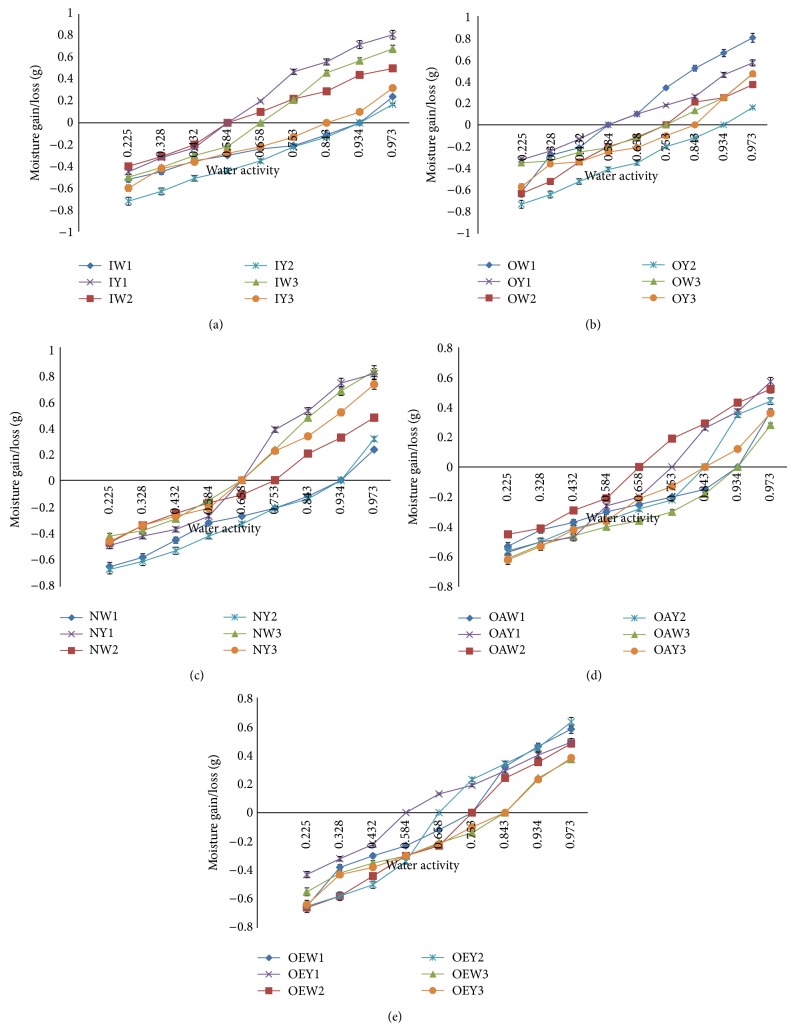
Water activity of garri samples (a) Ikpa, (b) Ogige, (c) Nkwo Ibagwa, (d) Obollo-Afor, and (e) Obollo-Eke (W: white; Y: yellow; 1–3 are different samples).

Figure 1 shows the water activity, moisture content relations and pattern of moisture migration in the various garri samples. The water activity values ranged from 0.587 to 0.934. In general, an increase in moisture content leads to a corresponding increase in water activity. Garri samples taken from the five markets followed similar trend of moisture migration at relative humidities lower or higher than their initial water activities. The samples from Nkwo Ibagwa and Ikpa markets had water activity below the critical level of 0.60 and so are considered to be safer for extended storage than samples from other markets (if such samples were to be stored for extended period). Water activity maxima and minima for fungal growth have been identified in stored food. The limiting level of water activity for growth of fungi is considered to be about 0.65. Only xerophilic species are likely to be able to grow at this level and even this will happen very slowly [41, 54]. Oxley [55] also stated that a water activity of 0.70 is low enough to ensure freedom from appreciable growth of fungi on most stored products. On the basis of the moisture content of the samples at point of collection, it can be deduced that the garri samples would be able to be stored for up to 6 months without mould infection [56] provided that moisture migration is prevented by storage in moisture proof packages. The reasons for the differences in the amount of absorbed moisture (percent) may be situated in differences in maturity and processing employed by the different manufacturers. Different producers toast garri to different levels of dryness depending on season, availability of wood fuel, and demand-supply dynamics among others. In general, during periods of glut garri is processed to extreme dryness to avoid spoilage before completion of sale. Similarly, garri intended for distant haulage tends to be toasted to very dry state to avoid spoilage before the sale. In view of the pattern of moisture sorption noted in this work, this practice may need to be subject to further study.

### 3.1. Moisture Sorption Isotherms of Garri

**Figure 2:**
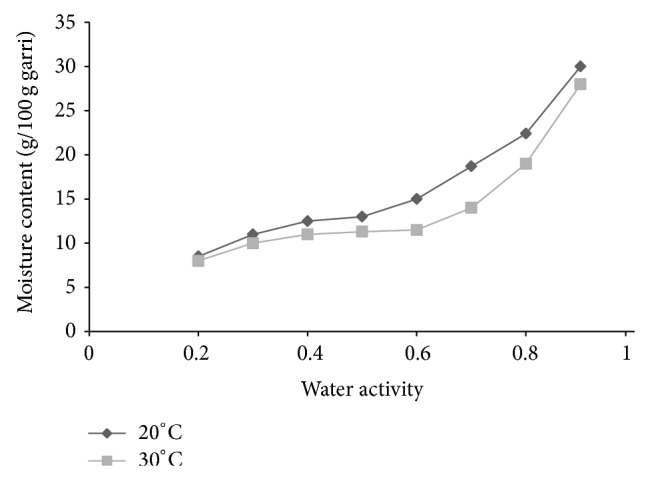
Moisture sorption isotherm of white garri at 20°C and 30°C.

**Figure 3:**
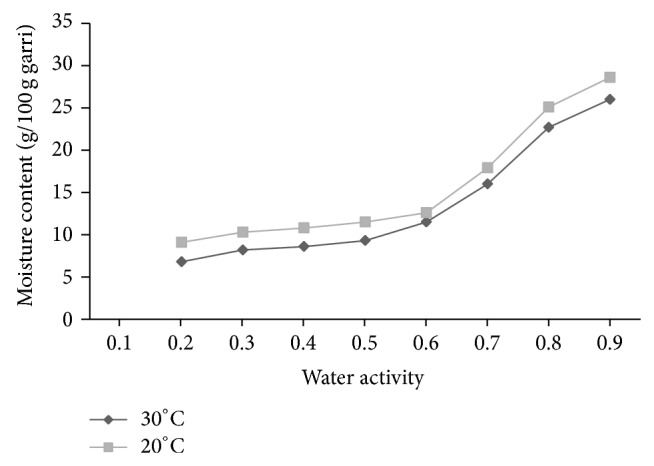
Moisture sorption isotherm of yellow garri at 20°C and 30°C.

Figures 2 and 3 show the moisture sorption isotherms of white and yellow garri, respectively, at 20° and 30°C. There was a general increase in the equilibrium moisture content (%) with increasing water activity (*a*_*w*_). This is due to the fact that the vapour pressure of water present in samples increased with that of the surroundings [57]. The equilibrium moisture content increased gradually up to water activity of about 0.4 and 0.5 before it increased rapidly (Figures 2 and 3). Although both exhibited type II sigmoidal behaviours the sorption characteristic for yellow garri was slightly different from that for white garri. The rate of change of moisture content with water activity for white garri was slightly higher than that for yellow garri at a given temperature. Thus, the processing technique has effects on the sorption characteristics. It is suggested more specifically that the lipid content of yellow garri modulated the water sorption behaviour of the final product. This, besides change in the nutritional content of the product due to changed lipid content, may be important in influencing mould ecology during long-term storage. Similar sigmoidal isotherm was reported in potato starch [52], dried cocoyam chip [41], cocoyam flour [24], and cassava flour [53]. According to Bolin [58] and Bandyopadhyay et al. [59], the type II isotherms are typical of foods high in carbohydrate. The result is understandable considering that garri is rich in carbohydrate (≥81.8%) [60]. Rockland [61] reported that products with such chemical composition exhibit gradual sloping isotherm at low water activity. The curves of the isotherms are gentle at water activity less than 0.5 where relatively low moisture was absorbed for a high increase in water activity. Above this level, high amount of water was absorbed for a small rise in water activity. Similar type II isotherms have been reported in rapeseed [30].

Water sorption was found to be marginally but not significantly temperature dependent. The higher the temperature, the lower the equilibrium moisture content (EMC) at constant water activity [62–65] (Figures 2 and 3). This implies that, at any water activity, garri becomes less hygroscopic with increase in temperature. Therefore, garri may be expected to absorb less moisture at higher temperatures. At fixed moisture content water activity will shift to higher values with increase in temperature. An increase in temperature at constant moisture content may cause a lowering of the isotherm curves. Similar behaviours have been reported in ginger [66], bambara groundnut powder [67], and castor seed [26]. This phenomenon would lead to an increase in water activity, thereby making the product more susceptible to microbial spoilage [58, 68]. This is explained by the higher excitation state of water molecule at higher temperature decreasing the attractive force between them [26, 69].

### 3.2. Mould Relation with Water Activity

Garri samples in the market are contaminated by an array of mould types. A total of 10 fungal species belonging to 7 genera of* Mucor*,* Penicillium*,* Cephalosporium, Aspergillus, Scopulariopsis, Rhizopus, *and* Paecilomyces *were isolated (Table 3). Previous reports have isolated moulds such as* Aspergillus, Penicillium, Rhizopus, Cladosporium, *and* Mucor *from garri during storage [12, 50, 70]. The processing of garri is associated with a high temperature (in excess of 90°C) critical control point and over a period long enough to inactivate any moulds associated with the fermentation of cassava or that could have prior entered the mash. Therefore, fungi isolated from garri are the result of postprocess handling and exposure.

At different water activity levels of 0.93, 0.8, and 0.7 the frequency of isolation of moulds corresponded to 100%, 60%, and 50%, respectively.* Mucor* spp. and* Aspergillus* spp. were the most frequently isolated (Table 3). Although this work did not seek to monitor changes in mould population over time, it is instructive that the frequency of isolation increased with water activity of samples. Ugwuanyi [41] reported* Aspergillus*,* Mucor,* and* Penicillium* at water activity range of 0.95–0.99 in dried cocoyam chips. Spoilage by filamentous fungi is one of the most important threats associated with processed and stored (dry) foods worldwide. Quality deterioration including discoloration, reduction in commercial value, and mycotoxin production has been linked to mould contamination of foods [12]. Various mycotoxins have been identified in foods and feeds contaminated by* Aspergillus species* [71, 72] and* Penicillium* spp. [73]. The fungi isolated from stored garri are a cause for concern for the safety of this food following storage under conditions that may encourage moisture absorption.

## 4. Conclusion

Moisture sorption isotherm of garri exhibited sigmoidal shapes described as type II, typical of dry carbohydrate rich foodstuff. The equilibrium moisture content of garri increased with water activity and decreased with increasing temperature. This study revealed the presence of various moulds in garri sold in the market particularly at higher moisture contents. This is exacerbated by the unwholesome but accepted mode of selling and distributing garri in open basins, trays, and mats in Nigeria market. These results highlight the need for more cautious handling of this product to ensure wholesomeness considering the variety of modes of consumption of the product and the need for enhanced shelf life. Packaging, particularly in moisture proof or modified atmosphere bags, is needed, especially in areas where humidity is high, in order to retain the low moisture content achieved by processing and prevent moisture migration.

## Figures and Tables

**Table 1:** Moisture content and water activities of white garri samples within 24 hours (0) of collection and following three (3) months of storage in hessian bags.

Sample source	Sample/moisture content (%)								
	WS1		WS2		WS3		Mean		
	0	3	0	3	0	3	0	3	% Change
Ikpa	9.50 ± 0.005^*^(0.60)	14.80 ± 0.002 (0.82)	11.20 ± 0.008 (0.67)	15.90 ± 0.002 (0.84)	9.30 ± 0.011 (0.61)	16.20 ± 0.002 (0.89)	10.00	15.63	56.33
Ogige main	11.10 ± 0.005 (0.67)	16.00 ± 0.002 (0.82)	10.00 ± 0.01 (0.66)	14.90 ± 0.002 (0.84)	11.20 ± 0.003 (0.75)	15.40 ± 0.002 (0.84)	10.77	15.43	43.27
Nkwo Ibagwa	10.80 ± 0.006 (0.60)	16.60 ± 0.002 (0.97)	11.80 ± 0.002 (0.72)	16.00 ± 0.002 (0.87)	9.90 ± 0.005 (0.65)	15.90 ± 0.003 (0.75)	10.83	16.17	49.31
Obollo-Afor	12.80 ± 0.002 (0.66)	15.60 ± 0.004 (0.93)	11.60 ± 0.003 (0.67)	14.60 ± 0.003 (0.84)	10.80 ± 0.002 (0.63)	16.00 ± 0.002 (0.91)	11.73	15.40	31.29
Obollo-Eke	9.90 ± 0.003 (0.65)	18.40 ± 0.002 (0.88)	10.60 ± 0.003 (0.69)	17.80 ± 0.002 (0.93)	11.70 ± 0.004 (0.84)	18.00 ± 0.002 (0.93)	10.73	18.07	68.41

^*^Figures in parentheses are the water activities of samples.

**Table 2:** Moisture content of yellow garri samples within 24 hours (0) of collection and following three (3) months of storage in hessian bags.

Sample source	Sample/moisture content (%)								
	YS1		YS2		YS3		Mean		
	0	3	0	3	0	3	0	3	% change
Ikpa	9.10 ± 0.006^*^(0.58)	17.80 ± 0.002 (0.75)	9.60 ± 0.005 (0.63)	17.10 ± 0.001 (0.93)	10.60 ± 0.005 (0.74)	18.10 ± 0.001 (0.93)	9.77	17.67	80.83
Ogige main	11.00 ± 0.01 (0.61)	14.30 ± 0.004 (0.65)	11.00 ± 0.005 (0.75)	18.40 ± 0.003 (0.97)	12.50 ± 0.006 (0.84)	17.80 ± 0.001 (0.87)	11.50	16.83	46.38
Nkwo Ibagwa	9.30 ± 0.006 (0.61)	17.40 ± 0.002 (0.75)	11.90 ± 0.001 (0.69)	18.20 ± 0.002 (0.93)	10.80 ± 0.003 (0.65)	16.80 ± 0.002 (0.75)	10.67	17.47	63.70
Obollo-Afor	12.10 ± 0.002 (0.75)	18.20 ± 0.003 (0.75)	11.80 ± 0.001 (0.74)	14.70 ± 0.004 (0.85)	12.50 ± 0.003 (0.84)	16.40 ± 0.002 (0.93)	12.13	16.43	35.45
Obollo-Eke	11.00 ± 0.003 (0.60)	17.00 ± 0.002 (0.75)	10.60 ± 0.003 (0.65)	18.20 ± 0.002 (0.89)	12.90 ± 0.001 (0.84)	18.60 ± 0.002 (0.94)	11.50	17.93	55.94

^*^Figures in parentheses are the water activities of samples.

**Table 3:** Occurrence of mould isolates in garri at different water activity levels.

Fungi	Range of water activity		
	0.93	0.8	0.7
*Mucor *spp.	2	1	1
*Penicillium nigricans *	1	1	—
*Cephalosporium *spp.	1	1	—
*Aspergillus niger *	2	1	—
*Aspergillus parasiticus *	1	1	—
*Aspergillus flavus *	2	1	—
*Scopulariopsis *spp.	1	—	1
*Penicillium digitatum *	1	—	1
*Rhizopus stolonifer *	2	—	1
*Paecilomyces farinosus *	1	—	2
% frequency of occurrence	100%	60%	50%
